# Extended brief intervention to address alcohol misuse in people with mild to moderate intellectual disabilities living in the community (EBI-ID): study protocol for a randomised controlled trial

**DOI:** 10.1186/s13063-015-0629-x

**Published:** 2015-03-25

**Authors:** Christos Kouimtsidis, Lucy Fodor-Wynne, Katrina Scior, Rachael Hunter, Gianluca Baio, Vittoria Pezzoni, Angela Hassiotis

**Affiliations:** iHEAR, Surrey and Borders Partnership NHS Foundation Trust, 1 Prince Regent Road, London, TW3 1NE UK; Division of Psychiatry, University College London, Charles Bell House, 67-73 Riding House Street, W1W 7EJ London, UK; Department of Clinical, Research Educational & Health Psychology, University College London, 1-19 Torrington Place, WC1E 6BT London, UK; Department of Primary Care and Population Health Research, University College London, Royal Free Hospital Rowland Hill Street, NW3 2PF London, UK; Department of Statistical Science, University College London, 1-19 Torrington Place, London, WC1E 6BT UK; Hertfordshire Partnership NHS Foundation Trust, Woodside Road, Abbots Langley, Watford, WD5 0HT UK

**Keywords:** Alcohol misuse, intellectual disabilities, brief intervention, AUDIT, RCT

## Abstract

**Background:**

There is some evidence that people with intellectual disabilities who live in the community are exposed to the same risks of alcohol use as the rest of the population. Various interventions have been evaluated in the general population to tackle hazardous or harmful drinking and alcohol dependence, but the literature evaluating interventions is very limited regarding intellectual disabilities. The National Institute for Health and Clinical Excellence recommends that brief and extended brief interventions be used to help young persons and adults who have screened as positive for hazardous and harmful drinking. The objective of this trial is to investigate the feasibility of adapting and delivering an extended brief intervention (EBI) to persons with mild/moderate intellectual disability who live in the community and whose level of drinking is harmful or hazardous.

**Methods/Design:**

The study has three stages, which include the adaptation of the Extended Brief Intervention (EBI) for people with intellectual disability, a single blind, randomised controlled trial of an individual Extended Brief Intervention to test the feasibility of the intervention, and a qualitative study that will assess the perceived acceptability and usefulness of the intervention. Fifty participants in total will be recruited from community intellectual disability services and social care or third sector organisations. The main outcome is a reduction in alcohol consumption measured by the Alcohol Use Disorders Identification Test.

**Discussion:**

Alcohol misuse is a relatively under-researched mental health problem in people with intellectual disabilities. Therefore, the study addresses both diagnostic issues and the delivery of a simple first stage intervention, which is available to the population of average intelligence and young persons in particular. The findings from the study will guide the preparation of a large-scale study to test whether this treatment is clinically and cost-effective in this population.

**Trial registration:**

ISRCTN58783633 (19 December 2013).

## Background

Alcohol is the most commonly used substance worldwide and contributes substantially to mortality, psychiatric and physical health morbidity and causes increased disease burden [1,2]. Despite the sparse literature regarding substance misuse in people with intellectual disabilities (characterised by an Intelligence Quotient (IQ) less than 70, developmental delay and adaptive deficits), there is increasing interest in studying such problems in this population. This is because most now live in the community and are more likely to be exposed to substances [3,4]. United Kingdom and United States population-based studies indicate that the prevalence of substance misuse in people with intellectual disabilities ranges from 0.5% and 2.5%, respectively, to as high as 25% for any substance in clinic samples [4-6]. Approximately 5% of the youth in drug and alcohol services have a degree of intellectual disability [1]. The most common substance that people with intellectual disabilities tend to use is alcohol, followed by cannabis and cocaine [2]. Aetiological factors that have been postulated to contribute to substance misuse in people with mild to moderate intellectual disabilities include hyperactivity, lack of assertiveness, low self-esteem, susceptibility to peer pressure, desire for social acceptance, social isolation, early onset of drinking and lack of example setting in childhood [1]. Substances may also be taken as a maladaptive way of relieving stress or developing relationships within local communities [7,8]. Those most at risk are young males with a mild intellectual disability or borderline intelligence who live independently and are less likely to engage in activities and/or to experience mental health problems [1].

Brief and extended brief interventions have been evaluated in the general population to tackle hazardous or harmful drinking and alcohol dependence. Despite the heterogeneity observed in the studies, a meta-analysis showed that participants generally have lower alcohol consumption at one year (mean difference: -38 grams/week, 95% CI: -54 to -23 and mean difference = -28, 95% CI: -62 to 6 grams/week, for brief and extended brief interventions, respectively) [9]. The same meta-analysis reports that loss to follow-up ranges from 10% to 70% but only three studies reported such rates. However, the literature evaluating any interventions targeted at people with intellectual disabilities is uncommon. This is partly due to the general lack of evidence based interventions in this field, but also to the belief that interventions that are suitable for typically functioning adolescents and adults are also suitable for those with mild-moderate intellectual disabilities without need for adaptation. This seems counterintuitive as people with intellectual disabilities have cognitive deficits that impair their ability to learn or generalise new learning, and therefore may require interventions to last longer, to include maintenance sessions, and to be supported to seek help and attend appointments. It is often reported that staff delivering interventions to the general population may not be skilled to provide such treatment to people with intellectual disabilities [8].

A variety of approaches have been tried to help people with mild to moderate intellectual disabilities and substance misuse, including education about the risks associated with substance misuse, behavioural modification, and the use of adapted materials from Alcoholics Anonymous or similar organisations, mostly delivered in group settings [10-16]. As a whole, these studies suggest that the capacity of people with intellectual disabilities to learn new information is enhanced by providing additional cues and using techniques such as modelling, videotaped vignettes and role-playing [15]. Often, sessions are augmented with coping skills lessons and assertiveness training [17]. However, the studies conducted to date are small, run in specialist single centre settings and are uncontrolled.

Nevertheless, two studies merit further attention; one is a study of three sessions of group motivational interviewing over two weeks conducted with seven offenders with intellectual disabilities in a medium secure unit [18]. The authors found that the participants showed increased determination to reduce drinking at the end of the treatment. The second study [15], was a 10-week evaluation of assertiveness training and modelling to educate about substance misuse and to help the participants (N = 84) to respond appropriately when offered substances in their social network. The authors found that knowledge of the risks associated with use of illicit substances increased at the end of the intervention, and this was maintained at six- months follow-up. The methodological limitations of the studies include the uncontrolled design, the small sample size, insufficient details of reporting, inclusion of several types of substances in the educational materials and the delivery in a specific environment that is different from community living.

The National Institute for Health and Clinical Excellence (NICE) (PH24; [19]) recommends that brief and extended brief interventions are used to help young persons and adults who have screened positive for hazardous and harmful drinking. The interventions recommended are based on motivational interviewing/enhancement techniques, and are delivered by trained professionals. Their aim is to reduce alcohol intake, reduce risk-taking behaviour and even to consider abstinence. The sessions are short at 30 minutes and follow-up is offered. At all times, individuals who are referred for treatment have their capacity to consent to it assessed and, where further treatment is indicated, referrals to secondary specialist services should be made.

We chose to evaluate the Extended Brief Intervention (EBI) in the current study because it is a widely used and evaluated treatment in the literature for hazardous and harmful drinking. It is a low-intensity intervention and can be delivered by trained professionals in the public and voluntary sector.

### Aims and objectives

The key research question is: Can we design a large scale, randomised controlled trial (RCT) that will answer whether EBI is more effective than usual care in helping adults with mild to moderate intellectual disability to reduce their alcohol intake?

The feasibility trial will compare the use of EBI with treatment as usual in the participating areas and will enable us do the following:

1. measure the number of eligible participants, including paid and family carers, willingness of clinicians to recruit participants, recruitment rate, loss to follow-up, adherence to the intervention, and standard deviation of the primary outcome measures. This will ultimately inform the sample-size calculation for a multicentre clinical trial.
2. determine the acceptability of randomisation to service users through its effect on dropout rates and qualitative interviews.
3. determine, through response rates to questionnaires, the appropriateness and the acceptability of the outcome measures to service users and their carers in order to explore the suitability of our chosen secondary outcome measures, that is, days abstinent, service use and health-related quality of life.
4. estimate the time needed to collect and analyse data
5. explore the utility of the health-related quality of life questionnaire in allowing the estimation of quality-adjusted life years in the sample.

We will also conduct qualitative interviews with service users, their carers, and service providers, to assess the acceptability of the treatment and to explore their experience of the treatment including any barriers and/or facilitators to taking part in the study. Findings from these interviews will enable us to refine the EBI.

## Methods/Design

The study corresponds to Phase 2 of the Medical Research Council (MRC) complex interventions guidelines, which guides the development of an intervention [20]. Therefore, the study has three stages that will be discussed below.

### Stage 1-Adaptation of the intervention

We will review the literature on EBI, and we will consult with service users and professionals about the number of sessions to be delivered and the materials to be used in each session. This is particularly important as people with intellectual disabilities have substantial cognitive deficits that require a longer duration of treatment, a mixture of materials, and the opportunity to test skills learnt over time [21]. So while the adapted intervention will include the existing treatment, such as a detailed assessment of drinking behaviour and the development of coping plans, it will be also include strategies to make engaging with the intervention easier, such as repetition of information and longer sessions.

The treatment manual will be tested with three service users (see Inclusion criteria below) in terms of acceptability and clarity, and the intervention will be refined accordingly. A similar approach has been used in a previous study [22]. The data from these individuals will not be included in the final analysis of the results.

### Stage 2-Feasibility study

#### Design

This will be a single blind, randomised controlled trial of individual EBI delivered as an adjunct to usual care for alcohol use disorders (AUD). Fifty participants, randomised to either the intervention arm or usual care, will be assessed at baseline, two months and three months. Research assistants carrying out assessments will be blind to the trial arm to which the participants are allocated.

Finally, qualitative interviews will be carried out at three months (see stage 3 below).

#### Setting

The study will be conducted within the Community Intellectual Disabilities Teams and social care services in three participating areas in England, UK, including urban and inner city locations. These teams offer specialist mental health, social care support and health services to adults with intellectual disability, as well as residential and supported living accommodation.

#### Participants

Fifty adults with mild to moderate intellectual disabilities aged 18 years and over, and who are known to professionals within the intellectual disabilities services as possibly having an alcohol problem, will be eligible to be referred to the study. Each participant should have a carer or family member prepared to accompany them to their sessions and complete the study instruments. Further justification for the sample size is available from http://www.nihr.ac.uk/CCF/RfPB/FAQs/Feasibility_and_pilot_studies.pdf.

Following informed consent, the participants will be assessed with the Wechsler Abbreviated Scale for Intelligence (WASI) [23] to ascertain their level of cognitive functioning (unless results from a previous cognitive assessment are available).

#### Recruitment

**Figure 1:**
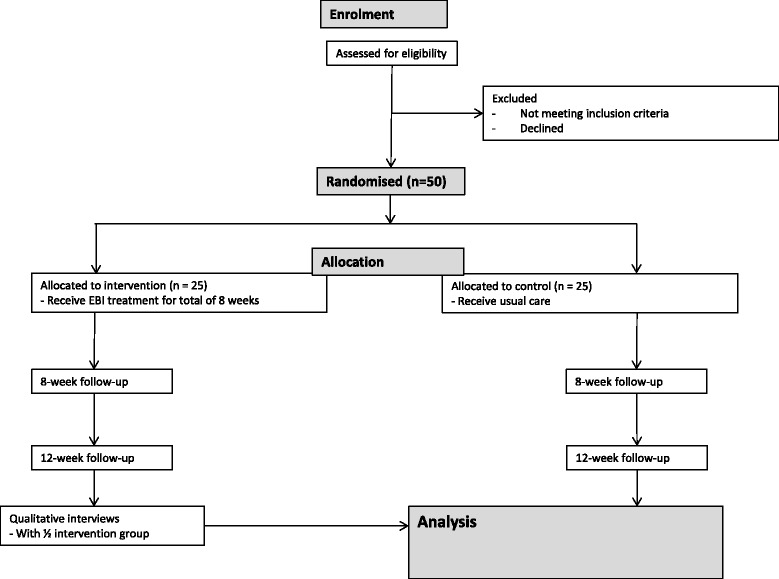
**Recruitment flow diagram.**

Recruitment of participants will be achieved through contacts and liaison with the intellectual disability services in the participating areas. Supported accommodation and social clubs in those areas will also be approached. Research assistants will work to increase publicity of the study by presenting it at all team meetings and submitting adverts for the study in organisational newsletters. Participant flow to the study is shown in Figure 1.

#### Inclusion criteria

Inclusion criteria are as follows:

1. AUDIT score >8 (providing the sum of questions 4, 5 and 6 is below 9). A score of 8 and above indicates hazardous or harmful drinking [24]. Questions 4, 5 and 6 specifically assess symptoms of dependence. The National Institute of Care and Clinical Excellence (NICE) [19] advises a reduction of scores in particular populations who may have lower prevalence of AUD, such as older people, females, and younger people. Local data, however, suggests that the prevalence of AUD in adults with mild/moderate intellectual disability in contact with services based on an AUDIT cut off of 8 is 22.5%, which is similar to the prevalence in the general population [4]. Therefore, a decision was made to adopt the same cut-off AUDIT score as in the general population.
2. Residents in the area within the last 12 months.
3. Full Scale IQ <70 (+/− 5% CI), which is the range for mild and moderate intellectual ability.

#### Exclusion criteria

Exclusion criteria are as follows:

1. severe to profound intellectual disabilities.
2. non-English speaking requiring interpreters.
3. in receipt of treatment for alcohol related problems in the last 12 months.
4. acute stage of severe and enduring mental illness.
5. poly-substance misuse including alcohol where the illicit substance, for example, cocaine/heroin/cannabis is the main problem.

#### Trial arms

The trial arms are as follows:

1. Intervention: EBI is modified as described in stage 1. It will be provided over five half-hour weekly sessions with a final one-hour session after eight weeks. It will be delivered by a trained therapist recruited from the NHS/private or third sector providing alcohol services in England. It is likely that the therapist may not have experience working with adults with intellectual disabilities; therefore, the research team members will provide training in the form of a one-day induction, including aspects of communication and how to overcome cognitive limitations in people with intellectual disabilities, interviewing techniques, use of the manualised treatment and guided reading to enhance clinical skills in working with this population. Furthermore, tapes of early treatment sessions will be reviewed by co-authors CK and KS. The therapist will be supervised regularly by CK, a consultant psychiatrist in addictions and trained in motivational interviewing.
2. Treatment as usual: Service users with mild to moderate intellectual disabilities who are identified as having AUD usually receive little specific support [25]; however, if problems escalate to dependence, they may be referred and managed jointly with secondary substance misuse services. Therefore, they may be offered non-specific counselling on improving coping abilities, pharmacological interventions where there are comorbid mental health problems or to achieve abstinence, inpatient admission if required, encouragement to attend community services for alcohol disorders, social support and nursing input if they experience any physical signs of malnutrition or epilepsy and other alcohol-related morbidity.

#### Outcome measures

The primary outcome is a reduction in alcohol intake measured by the modified Alcohol Use Disorders Identification Test (AUDIT) [24]. As there is limited experience of suitable measures for interventions for substance misuse in this population, the percentage of days of abstinence (PDAS) and percentage of days of heavy drinking (PDHD) will also be calculated. Definition of heavy drinking will be informed by a literature review [26]. These additional indices have been included in order to estimate which outcome may perform most reliably for a sample calculation for a definitive trial.

Secondary outcomes will be willingness to change, health status, service use, health-related quality of life and mental status.

#### Instruments

Instruments used in the trial will include the following:

1. AUDIT [24], which is a screening tool for identification of excessive drinking, an aid to brief interventions, and it has been used with people with mild to moderate intellectual disabilities. It will be administered at all treatment points.
2. Readiness to Change Questionnaire (RCQ) [27], which has been shown to have good validity and reliability. It contains 12 statements that must be read to the person with mild to moderate intellectual disabilities. It will be administered at baseline and three months.
3. Euro-QoL (EQ-5D-3 L); English version [28], which is a short questionnaire assessing general healthcare. This questionnaire has been used extensively in health research in the general population and more recently in studies of populations with intellectual disabilities. This will be administered at baseline and three months.
4. Clinical Outcomes in Routine Evaluation (CORE-LD) [29], which is an adapted version of the instrument for people with intellectual disabilities and measures self-reported psychological distress. It will be administered at baseline and three months.
5. Client Service Receipt Inventory (CSRI) [30], which is specifically adapted for the study to measure what services the client has been in receipt of in the past 6 months (for example, emergency room attendance, GP visits, benefits). It will be completed at baseline and three months for the previous three months. Data gathered by this tool will allow us to be more precise in recording the different services received by participants each study arm beyond the intervention and quantify the elements of ‘treatment as usual’.
6. Sociodemographic details at baseline.

All instruments will be completed by research assistants in face-to-face assessments with participants and paid or family carers. These assessments will take place at a convenient location. AH and KS will train Research assistants in interviewing techniques for people with intellectual disabilities, how to assess capacity to consent to research and how to administer the study instruments.

#### Randomisation and masking

Randomisation will be conducted by contacting an external researcher once the potential participant has consented to take part in the study and baseline data have been collected (class A method of randomisation). The research team statistician will generate the randomisation list. Any breach in blinding/unmasking, a common problem with single blind trials, will be monitored to ensure that the research assistants are not aware, as far as possible, to which group the individual was allocated. The researchers will make a note of which group he/she believes the participant has been allocated to for all participants and this will be compared to the actual allocations at the end of the study. The database management will be supervised by the PRIMENT Clinical Trials Unit and the study statistician GB.

#### Treatment fidelity

We will develop a short scale to measure how the treatment is delivered and which components are used in each session, guided by the literature [31]. Ten per cent of the audiotaped sessions will be rated by KS, who has no contact with the therapist. The therapist will be supervised regularly throughout the study duration by CK.

#### Statistical analysis

The analysis of the data will be mainly in terms of descriptive statistics and point and interval estimations with the express aim to inform a future multicentre trial. No hypothesis testing will be performed. We will compare drop-out rates in the intervention and treatment-as-usual arm, and means, medians, interquartile ranges, counts and proportions to estimate effect size will be calculated. Regression models allowing for participant clustering will be considered to examine the association between the primary outcome and sociodemographic and clinical predictors. The results will be presented with 95% confidence intervals.

#### Economic evaluation and analysis

The feasibility of collecting information for a full randomised control trial (RCT) cost-effectiveness analysis will be assessed, based on cost per gain in outcome of EBI compared to treatment as usual from a health and social care cost perspective. Costs will include the intervention, training and health and social care provision. Health and social care resource use, employment and the impact on carers will be costed using national published values. The feasibility of a cost-effectiveness analysis from a societal cost perspective will also be assessed. Descriptive statistics for these variables will be reported and an initial analysis will calculate 1) the mean incremental cost per reduction in days of heavy drinking and 2) the mean cost per quality adjusted life year (QALY) gained using the EQ-5D mapping algorithm. Previous studies in the general population have found that generic quality of life measures are not sensitive to reductions in alcohol misuse, particularly in hazardous drinkers [32]. We will test whether this is also the pattern in adults with mild to moderate intellectual disabilities and whether we can use these measures in a full trial using correlations, regression analysis and mapping. Some of the impact from long-term hazardous and harmful alcohol misuse can occur well into the future. Due to the short follow-up period, it is unlikely that the long-term impact will be captured within this trial, but this will require further investigation as part of a full trial using decision analytical models.

### Stage 3-Qualitative study

In order to assess the perceived acceptability and usefulness of the intervention, 50% of service user and carer dyads allocated to the EBI arm of the trial, will be interviewed at end of treatment (that is, 12 weeks). The interviews will be completed by the two service users who are supported to contribute to the study reference group (part of the trial management group), with support from the research assistants. Both service users and the research assistants will be trained in how to collect qualitative data and conduct the interviews. We will include participants who may have dropped out of treatment, if at all possible, in order to ascertain their perceptions of the intervention and their reasons for dropping out. Interviews will be audiotaped and analysed using content analysis and/ or thematic analysis [33,34].

### Ethical and research and development reviews

The study has been approved by the NRES Committee South Central - Berkshire (REC reference 13/SC/0143).

All study personnel will comply with the MCA 2005 [35] and published research governance guidelines. We anticipate that all participants will have capacity to consent and sufficient verbal communication skills to take part in the treatment and in the qualitative interview. All patient information sheets and consent forms will be in easy-read versions, and informed written consent will be obtained from all participants. As this is a non-invasive intervention, we do not anticipate any adverse events, but we will follow safety reporting guidance issued by the National Research Ethics Service (UK) for studies except clinical trials of investigational medicinal products.

The study has been approved by the Research and Development departments of the following organisations: Hertfordshire Partnership NHS Foundation University Trust, Camden and Islington NHS Foundation Trust, Surrey and Borders Partnership NHS Foundation Trust.

### Patients and public involvement

We consulted with a service user group based in one of the research areas on two different designs for the study, one being the current version and the other a feasibility study of contingency management. The latter is defined as treatment based on principles of behaviour modification and aims to incentivise and then reinforce changes in behaviour with the aid of vouchers, privileges, prizes or modest financial incentives that are of value to the client (https://www.nice.org.uk/guidance/cg51/chapter/appendix-c-contingency-management-key-elements-in-the-delivery-of-a-programme). Based on the various issues identified as potential difficulties in each option, we made the decision to start from a simpler project that has yet to be tested in this population. An easy-read version of the project proposal was presented to a service users’ group for advice and comments on how to present the study and describe the intervention. Two service users and their carers contribute to the trial steering committee and will conduct the qualitative interviews as described earlier. The service users will be involved in the interpretation of the interviews, write up the findings in an accessible format and disseminate them locally and nationally.

## Discussion

The study will provide invaluable information on several aspects of alcohol and other substance misuse patterns in people with intellectual disability. Although not an epidemiological study per se, it will reveal important elements of treatment pathways and support needs for those individuals. Such information is currently either not available or mostly derived from clinic populations. Our sample will be drawn from a wide variety of community care.

Furthermore, there is limited literature on use of alcohol instruments such as AUDIT and RCQ in people with intellectual disabilities; therefore, the adapted tools used in this project will be particularly useful for further research but also in clinical practice in this field.

Most importantly, there is emerging debate about the impact of brief interventions delivered in primary care on alcohol problems [36]. Current literature suggests that they indeed reduce consumption, but there is little information on the content and fidelity of the intervention in published studies.

In carrying out the study, we will need to overcome generic difficulties in research in adults with substance misuse who may be not only be hard to reach but also unwilling to commit to treatment or may drop out. Access to participants may depend on local configurations of services, and therefore, the recruitment strategy will be crucial in carrying out the feasibility study. Therefore, our work will be sufficiently novel to contribute not only to the field of intellectual disabilities but to the existing research in this particular topic.

## Trial status

At the time of the manuscript submission the trial is still recruiting new participants.

## Abbreviations

AUDIT: Alcohol Use Disorders Identification Test
EBI-ID: extended brief intervention-ID
ID: intellectual disabilities
RCT: randomised controlled trial
IQ: intelligence quotient
